# Dual Promoters Improve the Rescue of Recombinant Measles Virus in Human Cells

**DOI:** 10.3390/v13091723

**Published:** 2021-08-30

**Authors:** Soroth Chey, Juliane Maria Palmer, Laura Doerr, Uwe Gerd Liebert

**Affiliations:** Institute of Virology, University Hospital Leipzig, Johannisallee 30, 04103 Leipzig, Germany; Soroth.Chey@medizin.uni-leipzig.de (S.C.); juliane@stos.ch (J.M.P.); Doerr.laura@gmx.net (L.D.)

**Keywords:** measles virus, reverse genetics, negative-strand RNA virus, recombinant virus, multivalent vaccines, oncolytic vectors, mononegavirales

## Abstract

Reverse genetics is a technology that allows the production of a virus from its complementary DNA (cDNA). It is a powerful tool for analyzing viral genes, the development of novel vaccines, and gene delivery vectors. The standard reverse genetics protocols are laborious, time-consuming, and inefficient for negative-strand RNA viruses. A new reverse genetics platform was established, which increases the recovery efficiency of the measles virus (MV) in human 293-3-46 cells. The novel features compared with the standard system involving 293-3-46 cells comprise (a) dual promoters containing the RNA polymerase II promoter (CMV) and the bacteriophage T7 promoter placed in uni-direction on the same plasmid to enhance RNA transcription; (b) three G nucleotides added just after the T7 promoter to increase the T7 RNA polymerase activity; and (c) two ribozymes, the hairpin hammerhead ribozyme (HHRz), and the hepatitis delta virus ribozyme (HDVrz), were used to cleavage the exact termini of the antigenome RNA. Full-length antigenome cDNA of MV of the wild type IC323 strain or the vaccine AIK-C strain was inserted into the plasmid backbone. Both virus strains were easily rescued from their respective cloned cDNA. The rescue efficiency increased up to 80% compared with the use of the standard T7 rescue system. We assume that this system might be helpful in the rescue of other human mononegavirales.

## 1. Introduction

The measles virus (MV) is an enveloped, non-segmented, negative-sense, single-stranded RNA virus. It carries a single copy of the genome, which is 15,894 bp in length. The genome of MV encodes six major structural proteins, i.e., nucleocapsid protein (N), phosphoprotein (P), matrix protein (M), fusion protein (F), hemagglutinin (H), and the large protein (L). Two additional nonstructural proteins, V and C, are produced from the P gene by RNA editing. The viral genome is encapsidated by N, P, and L proteins forming the ribonucleoprotein complex (RNP), surrounded by M protein. H and F proteins mediate virus attachment and fusion, respectively [1]. Attenuated MV is one of the most effective and safe vaccines available. Many laboratories worldwide are intensively researching MV as a safe vector candidate for developing multivalent vaccines and highly promising oncolytic vectors in cancer therapy. The reasons for the increased interest in MV are plenty; for example, its proven safety and efficacy in humans for over 60 years, its lack of genotoxicity (it replicates only in the cytoplasm and does not integrate into the genome of host cells), its long-lasting immunogenicity, its high genetic stability compared to other RNA viruses, its oncolysis in tumor cells, and the many technological opportunities offered by MV reverse genetics [2].

Reverse genetics is a molecular biology method for creating a new virus from its cloned cDNA. This technique facilitates the understanding of viral biology and encourages the further development of novel vaccines and vectors for gene delivery. The first recovery of a non-segmented negative-strand RNA virus entirely from cloned cDNA was achieved in 1994 for the rabies virus [3]. Since then, other non-segmented negative-strand RNA viruses have been recovered from cloned cDNA [4,5,6].

The original reverse genetics system for rescuing the MV is based on the co-transfection of two plasmids, the full-length cDNA plasmid p(+) MV323-eGFP and the helper plasmid for the expression of the large (L) protein pEMC-La, into the helper cell 293-3-46, which stably expresses the MV proteins N, P, and the T7 RNA polymerase [7]. The viral antigenomic RNAs and the viral N, P, and L RNA are transcribed by T7 RNA polymerase in the cytoplasm. The antigenomic RNAs are then encapsidated by N, which associates with the viral polymerase L and its co-factor P to form biologically active ribonucleoprotein (RNP) complexes. These RNP complexes can be used for transcription and replication, ultimately resulting in the rescue of infectious viruses [7]. This original reverse genetics system allows repeated recovery of MV vaccine strains [8], wild-type [9], and even gene-deleted virus [10,11]. However, this method is inefficient. Several attempts were made to increase the efficiency of MV rescue. These include modifying the use of other susceptible cells (CHO-hSLAM) [12], following the use of recombinant modified vaccinia virus Ankara (MAV-T7) [13,14] or vaccinia virus Lister vaccine strain [15] to increase sufficient T7 RNA polymerase expression, or involving the use of host polymerase II promoters (CMV) to drive viral RNA synthesis [16,17]. However, the efficiency of the recovery remained relatively low, and there is a need for improvement. Rescue efficiencies in the systems with T7-expressing vaccinia virus are high, though laborious cleaning steps are required. Improvement of the reverse genetics system can involve different strategies, such as using multiple promoters to improve the efficiency of viral RNA expression in the nucleus and cytoplasm [18]. In this system for recovery of the influenza A virus, two promoters—the RNA polymerase II promoter (CMV) and the T7 promoter—are used. The CMV promoter is used for the transcription in the nucleus, whereas the T7 promoter is responsible for the transcription in the cytoplasm [18]. The exact 3′ and 5′ ends of the antigenome RNA are critical for successfully assembling the first ribonucleoprotein (RNP) complexes. More recently, optimized ribozyme sequences with an enhanced cleavage activity were developed [19]. These ribozymes are the autocatalytic activity of an 85-nucleotide-long hepatitis delta virus ribozyme (HDVrz) and an autocatalytic Hammerhead ribozyme (HHrz), which truncate the exact termini of the rabies virus antigenome RNA. They showed that the combination of the optimized HHrz and HDVrz increased rabies virus rescue efficiency more than 100-fold. Today, there is no existent reverse genetics plasmid system for MV that uses both combinations: dual promoters on the same template and high cleavage activity ribozymes HHrz and HDVrz at both ends of the RNA [19].

In the present study, we constructed a plasmid platform that uses two promoters, the RNA polymerase II promoter (CMV) and the bacteriophage T7 promoter, in a uni-direction. In this construct, three G nucleotides were added just after the T7 promoter to enhance the T7 RNA polymerase activity, and two optimized ribozymes HHrz and HDVrz sequences were used to cut the exact termini of the antigenome RNA of MV. Full-length antigenome cDNA of MV, either the wild type IC323 strain or the vaccine AIK strain, was inserted into this plasmid backbone. Both virus strains were rescued efficiently from their cloned cDNA. This reverse genetics platform can increase the rescue efficiency up to 80% compared to the standard T7 rescue system.

## 2. Materials and Methods

### 2.1. Cells and Plasmids

Vero cells expressing human SLAM (Vero/hSLAM) [9] and the helper cell line 293-3-46 [7] constitutively expressing T7 RNA polymerase as well as measles N and P proteins were maintained in DMEM medium supplemented with 10% fetal calf serum (FCS), penicillin-streptomycin (100 U/mL) (Gibco, Waltham, MA, USA), and geneticin G418 (0.5 mg/mL) (Biochrome, Berlin, Germany), respectively.

The plasmid p(+)MV323-eGFP [20,21] was used as a template for generating MV wild type (MV wt) antigenome sequence. The plasmid p(+)MVAIK-eGFP [8,22] was used as a template for producing the MV AIK-C antigenome sequence.

### 2.2. Construction of the Vector Backbone Plasmid pT-HHrz-HDVrz

The plasmid pCi-neo-CAV1 [23] was used as the template for the PCR with the forward primer trailer_HDVrz-pCi-s and the reverse primer T7_HHrz_leader_as (Table S1). The PCR amplificates were gel-purified and blunt-end ligated with T4 ligase and then transformed into *E. coli* NEB-5-alpha. The resulting plasmid pCi-T7-HHrz-HDVrz was then subcloned into a low-copy plasmid piRFP [24] by digestion of the plasmid pCi-HHrz-HDVrz with restriction enzymes SalI and SacI (Figure S1B). The fragment containing the cassette CMV-intron-t7-HHrz-HDVrz was gel-purified. Similar restriction enzymes SalI and SacI were used to digest the low copy plasmid piRFP (Figure S1C). The vector backbone fragment was gel-purified. The two gel-purified fragments (the piRFP backbone DNA fragment and the cassette chimeric intron-t7-HHrz-HDVrz fragment) were ligated. The upcoming plasmid piRFP-HHrz-HDVrz (Figure S1D) was next digested with restriction enzymes NheI and HindIII, and the remaining vector backbone fragment (4470 bp) was gel-purified, overhangs were trimmed with T4 DNA polymerase, and the blunt ends of the plasmid were ligated using T4 ligase.

### 2.3. Construction of Full-Length MV cDNA

The DNA fragment of the vector backbone plasmid pT-HHrz-HDVrz was produced by a PCR using primer pair: MV_Trailer_s and MV_Leader_as (Table S1). This PCR fragment was gel-purified and used for inserting the MV antigenome. The plasmid p(+)MV323-eGFP [19] or pMVAIK-GFP [20], which contain the full-length antigenome cDNA of MV IC-B or MV AIK-C, was used as the template for the generation of the MV antigenome fragments. The primers were designed for covering the whole antigenome of MV (Table S1), and three PCRs fragments (F1, F2, and F3) were amplified using corresponding primer pairs (Table S1 and Figure S2). These three PCR fragments were gel-purified and assembled sequentially in the vector backbone plasmid pT-HHrz-HDVrz fragment between the HHrz and the HDVrz ribozymes by using Gibson Assembly kit (NEB, Frankfurt am Main, Germany).

### 2.4. Rescue of the MV from Cloned cDNA

Recombinant MV IC323-eGFP and MV AIK-eGFP were recovered from the full-length cDNA original plasmids p(+) MV323-eGFP and p(+) MVAIK-eGFP or the newly cloned plasmids pT(+)MV323-eGFP and pT(+)MVAIK-eGFP using the reverse genetics method as reported previously with a modification [7,9,25]. Briefly, 2 × 10^5^ helper cells 293-3-46 in each well of a 6-well plate were transfected with 5 µg full-length-genome plasmids and two helper plasmids, 10 ng of pEMC-La plasmid expressing the measles large (L) protein [7] and 250 ng of pCIAN01 plasmid expressing the measles N protein [8] using calcium phosphate transfection. One day after transfection, the cells were heat-shocked in a water bath at 44 °C for 1 h. Next, 24 h after the heat-shock, the helper cells were overlaid onto Vero/hSLAM cells in a 6-well plate.

### 2.5. Growth Analysis

A multiple-step growth kinetics study was performed to assess the difference in replication of the viruses rescued from cloned cDNA plasmids (origin and new). Vero/hSLAM cells (2 × 10^5^/well) in 6-well plates were infected with MV at a MOI of 0.01 for 2 h at 37 °C. The supernatant was removed and replaced with an equal volume of fresh medium. Virus titers were determined on Vero/hSLAM cells by 50% tissue culture infective dose (TCID_50_) titration [26].

### 2.6. Detection of Viral Proteins

Immunohistochemistry assays were used for the detection of MV N antigens in transfected cells. Vero/hSLAM cell monolayers grown on glass coverslips were infected with MV at a MOI of 0.01. Two days post-infection (dpi), the cells were fixed in 4% paraformaldehyde for 10 min, washed with PBS, and blocked for 1 h at RT with 10% normal goat serum (Jackson Immuno Research, Baltimore, MD, USA) in PBS—0.2% triton X-100. After blocking, the coverslips were incubated with primary antibodies F227 mouse-anti-MV-N ((F227/3, produced in-house), 1:5 dilution in PBS 0.3% triton X-100, overnight at 4 °C. The glass coverslips were washed three times with PBS 0.2% triton X-100 and incubated with fluorescence-labeled secondary antibodies (Alexafluor 546 donkey-anti-mouse, Invitrogen, Waltham, MA, USA) 1:500 dilution in PBS 0.2% triton X-100 for 1 h. After washing, the glass coverslips were mounted using Entellan fluorescence mounting medium (Merck, Darmstadt, Germany) and viewed under a fluorescent microscope.

## 3. Results

### 3.1. Construction of the Vector Backbone Plasmid and Cloning of the Full-Length cDNA of MV

**Figure 1:**
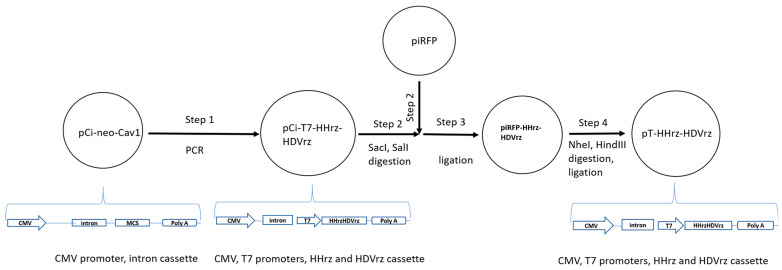
Cloning schemata of the vector backbone plasmid pT-HHrz-HDVrz.

The vector backbone pT-HHrz-HDVrz was generated by molecular cloning methods through several steps as schematically described in Figure 1. The aim was to create a cassette of two promoters and the ribozymes at both ends of the inserted MV antigenome. For this purpose, the plasmid pCi-neo-CAV1 contains a cassette of CMV promoter and the intron and was used as the template for the PCR with the designed primers containing the ribozymes and MV’s leader and trailer sequences. The resulting plasmid (Figure 1, step 1) was named pCi-T7-HHrz-HDVrz and was then used to be subcloned into a low copy plasmid piRFP by digestions of both plasmids with restriction enzymes SalI and SacI (Figure 1, step 2). The resulting plasmid clone (Figure 1, step 3) was named piRFP-HHrz-HDVrz and confirmed by sequencing. In the plasmid piRFP-HHrz-HDVrz, there is a partial fragment of the iRFP gene left (Figure S1D). The plasmid piRFP-HHrz-HDVrz was digested with restriction enzymes NheI and HindIII to remove the remaining iRFP fragment (408 bp). The resulting remaining vector backbone was ligated into a new plasmid pT-HHrz-HDVrz (Figure 1, step 4) and confirmed by sequencing. This pT-HHrz-HDVrz plasmid contains a cassette of two promoters CMV and T7, where three G residues were placed directly downstream of the T7 promoter sequences. The HHrz sequences were designed according to the leader sequences of MV, where the hairpin structures promote correct folding and exact cleavage at the 5′ end of the MV sequences. At the 3′ end of the cassette, the HDVrz provides exact cleavage at the 3′ end of the RNA [17]. The HDVrz was placed between the MV trailer sequences and the SV40-polyA-terminator (Figure S1E).

This vector backbone pT-HHrz-HDVrz was used as a vector backbone for inserting the full-length cDNA antigenome of MV between the HHrz and HDVrz sequences in the next step.

**Figure 2:**
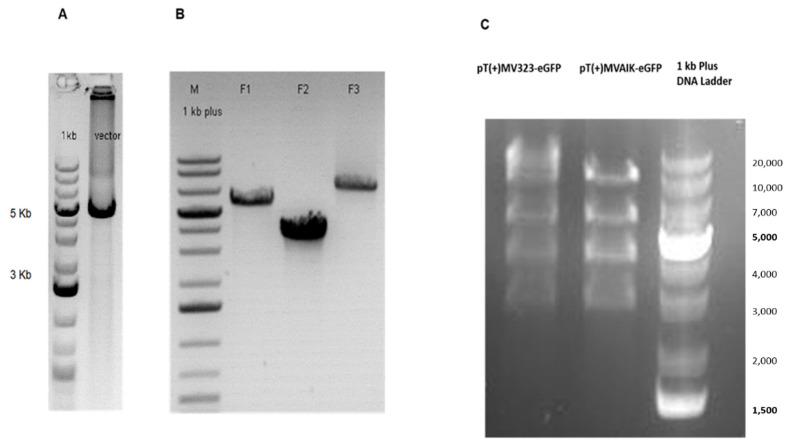
Schematic diagrams of construction of full-length antigenome MV plasmids. (**A**) The vector backbone V was generated from the PCR using shuttle plasmid template and primers MV_Trailer-s and MV_Leader-as. (**B**) Full-length MV plasmid was cloned by ligation of vector backbone V and three PCRs fragments generated from the donor full-length MV antigenome template plasmid p(+)MV323-eGFP or p(+)MVAIK-eGFP. (**C**) The plasmid pT(+)MV323-eGFP and pT(+)MVAIK-eGFP clone were analyzed by restriction digestion with BamHI.

As shown in Figure 2A, PCR products V of the vector backbone with expected sizes were verified (4478 bp). The full-length cDNA of either the MV IC323 strain or the MV AIK-C vaccine strain was generated by three PCR runs. These three PCR fragments (F1 = 5672 bp, F2 = 3682 bp and F3 = 7510 bp) of full-length MV antigenome were gel-purified and analyzed by Agarose gel electrophorese (Figure 2B). The fragments were used as components of the Gibson Assembly. The resulting plasmid was confirmed by sequencing with the primers listed in Table S2 and restriction digestion analysis (Figure 2C) and designated as pT(+)MV323-eGFP or pT(+)MVAIK-eGFP.

### 3.2. Efficient Recovery of Recombinant MV

**Figure 3:**
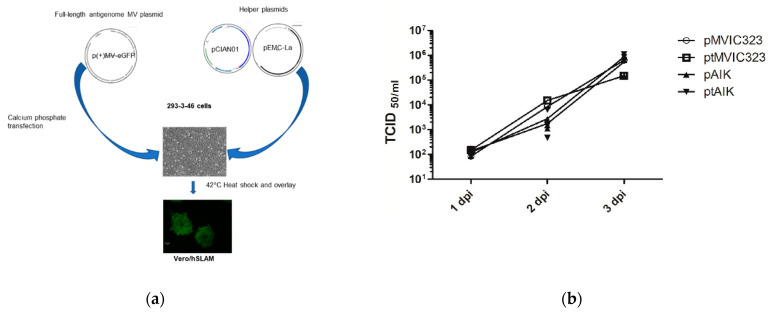
Rescuing the MV from cDNA and growth curve kinetic: (**a**) Schemata of the reverse genetics protocol as described in the method; (**b**) multiple-step growth kinetics of four recombinant MVs in Vero/hSLAM. pT_IC323 (rescued from plasmids pT(+)MVIC323-eGFP), pT_AIK (rescued from pT(+)MVAIK-eGFP), IC323 (rescued from p(+)MV323-eGFP), and AIK (rescued from p(+)MVAIK-eGFP) were analyzed on Vero/hSLAM at different time points.

To test whether the newly constructed plasmids can improve virus rescue efficiency, recombinant MVs were rescued from four plasmids: two were original plasmids, and the other two were constructed in this paper. The helper cells 293-3-46 were transfected with each of the full-length plasmids listed in Table 1, together with the helper plasmids coding for N and L proteins, using the standard reverse genetics protocol (Figure 3a) as described in the method. At 3 to 5 days after the overlay, one or more green syncytia cells could be visualized under the fluorescent microscope due to eGFP expression. The green syncytia were evaluated under a fluorescence microscope and by immunofluorescence assays. The green eGFP syncytia corresponded with the red fluorescent MV N proteins (Figure S3). Therefore, each green-positive syncytia was considered to represent a positive rescue event. The numbers of syncytia were counted under a fluorescent microscope in each well of a 6-well plate to estimate the number of recovery events.

The rescue event of the MV from the original plasmid was rare if any. Only one rescue event was found in one well after five independent rescue experiments (total 30 wells, Table 1). In contrast, the newly constructed plasmids generated more rescue events (2 to 6 events/well) and boosted the rescue efficiency up to 80% (Table 1). To rule out the possibility that variable rescue efficiency is due to defects in the virus genome, the growth kinetics of the rescued MVs in Vero/hSLAM cells were compared in multiple-step growth kinetics (Figure 3b). Infection of Vero/hSLAM cells at a MOI of 0.01 revealed identical growth kinetics. This showed that the improved rescue efficiency was due to the enhanced components in our plasmid.

## 4. Discussion

We developed a reverse genetics plasmid platform that follows several improvements to enhance the recovery of MV in the human cells: (1) the RNA polymerase II (CMV) promoter and the bacteriophage T7 RNA polymerase promoter were placed on the same plasmid to increase viral RNA transcription. (2) An intron was inserted downstream of the CMV promoter to stabilize the RNA and transport it to the cytoplasm. (3) Three G nucleotides were added directly downstream of the T7 promoter to enhance T7 RNA polymerase. (4) Two ribozymes, HHrz and HDVrz, flanking the full-length cDNA termini, were used for cleavage, leading to exact antigenome RNA termini.

The transcription of the full-length antigenomic MV RNA and three helper proteins N, P, and L in the standard reverse genetics system was carried out under the control of T7 RNA polymerase. The source of the T7 RNA polymerase was the helper cell line 293-3-46, which constitutively expressed T7 RNA polymerase, MV N, and P proteins [7]. Although this standard method led to the rescue of MV from the cDNA, we noted that the rescue efficiency was low (Table 1 and [12]). There are three main reasons for this low recovery efficiency in the standard reverse genetics system. First, the helper cell line 293-3-46 may only express a low level of T7 RNA polymerase, which leads to insufficient transcription of the antigenome RNA and the MV N and P proteins. Second, the lack of the three G nucleotides just after the T7 promoter leads to lower T7 RNA polymerase activity. Third, the 3′ end of the full-length RNA may not be effectively cleaved by the standard HDVrz.

To improve transcription of the viral RNA, two promoters, the cellular RNA polymerase I and RNA polymerase II promoter, were used to transcribe the viral RNA. These two promoters could raise the chance of transcriptions of the virus gene by cellular RNA polymerase I and II in a single cell [27,28,29].

In our novel plasmid platform, two promoters, the cytomegalovirus (CMV) and the T7 promoter, were used. We first constructed a rescue system with only the CMV promoter [30] and compared it with the original plasmid p(+)MV323-eGFP, which only had the T7 promoter. The result of the rescue system with CMV promoter only was as weak as the original plasmid p(+)MV323-eGFP. Therefore, we built an additional T7 promoter downstream of the CMV promoter. The transcription and replication of MV RNA occur mainly in the cytoplasm, so the RNAs transcribed by T7 polymerase were directly used for forming the RPN complex. However, the viral RNA expressed by the cellular RNA polymerase II in the nucleus should be transported from the nucleus to the cytoplasm. To address this problem, an intron sequence downstream of the CMV promoter was inserted to ensure splicing and capping, thus enabling efficient export to the cytoplasm and a stabilization of the RNA [29,30,31,32,33].

To increase the efficiency of T7 RNA polymerase transcription, three guanidine residues were added just after the T7 promoter sequence. These three guanidine residues formed an optimal initiation sequence for T7 RNA polymerase [33,34,35]. However, adding three extra nonviral G nucleotides could violate the rule of six [36] and hamper the RNP formation. One strategy used to cope with this problem in the past has been the insertion of a hammerhead ribozyme (HHrz) after the GGG of the T7 promoter, creating an exact 5′ end and improving the rescue efficiency [37,38].

The critical factor for efficient recovery of the negative-strand RNA virus is the exact termini of the resulting transcribed full-length RNA. More recently, Ghanem et al. designed an optimized HDVrz ribozyme with an enhanced cleavage activity compared with the standard HDVrz ribozyme [19]. They added two ribozymes at both ends of the cDNA sequences, which could produce exact termini of viral antigenome RNA and increased rabies virus rescue efficiency by more than 100-fold. Our paper followed a similar strategy as published by Seki et al. [39], who also used Hammerhead ribozyme (HHRz) and HDV ribozyme (HDVrz) to determine the exact size of full-length viral RNA. However, Seki et al. used the reverse genetics system based on only the T7-Promoter and the hamster BHK-T7 cell line. When the authors used the original plasmid p(+)MV323-eGFP and the plasmid containing the SI strain rSI-AcGFP to rescue the virus by the reverse genetics system reported by Radecke et al. [7], as in our work, it failed to function until they used the vaccinia virus to express the T7 RNA polymerase [39]. Beaty et al. applied the same strategy with the optimized T7 promoter and the two ribozymes at both ends of the transcribed RNA, but this system was only tested with the rescue cell types BSR-T7/5 cells, which were derived from the hamster cell lines BHK-21 [40]. It has not been tested with other rescue cell types, such as 293-3-46 cells, so the reverse genetics system may only work with those rescue cell types. With the same hamster rescue cell types BSR-T7/5, the research group of Muñoz-Alía et al. used two promoters, CMV and T7, which led to higher efficiency of virus rescue [41]. Our result matches that of Muñoz-Alía et al. but using the human rescue cell types 293-3-6. Moreover, the rescue cell types BHK-T7 cell and BSR-T7/5 are from hamsters and are not human cells. Therefore, our system adapted to the human cells 293-3-46 is more suitable for the MV than the Seki system, which performs rescue in the hamster cells that are not susceptible to the MV growth.

In conclusion, an improved reverse genetics plasmid platform was engineered with several advantages over standard reverse genetics methods. Using this plasmid platform, recombinant MVs were rescued from their cloned cDNA with high recovery efficiency [42]. The rescue efficiency increased up to 80% compared with the use of the standard T7 rescue system. It would be interesting to include this plasmid platform into the reverse genetics system of other mononegavirales and find out whether this plasmid platform might improve the recovery of those viruses.

## Figures and Tables

**Table 1:** Comparison of recovery of MV from different plasmids. Four plasmids were used to rescue MV using the standard reverse genetics protocol. Five independent reverse genetics experiments were done every plasmid using 293-3-46 cells on 6-well plates for transfections and Vero/hSLAM for overlay and co-culture. Syncytia were counted on Vero/hSLAM to estimate the number of recovery events.

Plasmid	Number of Recovery Events/Per Well (5 Independent Experiments)	Number of Positive Wells (n = 30 Wells)	Percentage of Positive Wells
p(+)MV323-eGFP [12]	0		
	1		
	1	3	10%
	0		
	1		
p(+)MVAIK-GFP [8]	0		
	0		
	0	1	3%
	0		
	1		
pT(+)MVAIK-GFP [this paper]	4		
	2		
	6	21	70%
	4		
	5		
pT(+)MV323-eGFP [this paper]	6		
	3		
	6	24	80%
	5		
	4		

## Data Availability

The dataset analyzed for the current study is available from the authors upon reasonable request.

## Supplementary Materials

The following are available online at www.mdpi.com/article/10.3390/v13091723/s1, Table S1: Primers used to generate plasmid back bone; Table S2: Primers used to sequence the MV plasmids; Figure S1: Vector maps of the plasmids; Figure S2: Schematic diagrams of construction of full-length MV cDNA; Figure S3: Immunohistochemistry for detecting MV N protein.
