# Supplementary material for: Dual Promoters Improve the Rescue of Recombinant Measles Virus in Human Cells
Source: Viruses. 2021 Aug 30;13(9):1723. doi: 10.3390/v13091723 (PMC8471996; doi:10.3390/v13091723)
Supplement: Supplementary file 1 [file viruses-13-01723-s001.zip › viruses-1334557-supplementary.pdf]

Supplementary Material

Table S1: Primers used to generate plasmid back bone. The MV antigenome cDNA sequence is displayed in underlined lowercase italics letters, the ribozyme HDVrz is shown in capital letters and the partial pCi-neo-CAV1 vector is indicated in bold lowercase letters). The reverse primer R1 has a hammerhead ribozyme sequence (HHRz) , the T7 promoter sequence and leader sequence of the MV antigenome. Numbers in the nucleotide location indicate the position on the MV IC-B sequence (accession no.: AB016162.1).

**Name**	**Nucleotide location**	**sequence (5´--->3´)**
trailer_HDVrz-pCi-s	15874 - 15894	*gtttctattcccagctttgtctggt*GGGTCGGCATGGCATCTCCACCTCCTCGCGGTCCGACCTGGGCATCCGAAGGAGGACGCACGTCCACTCGGATGGCTAAGGGAGAGCCA**ctagagtcgacccggg**
T7_HHrz_leader_as	1 -21	*ctatccttacccaactttgtttggt*GACCCGGTACGCCGGGTTTCGTCCTCACGGACTCATCAGACCAAAC**cctatagtgagtcgtattaa**
MV_Trailer_s	15874 - 15894	GTTTCTATTCCCAGCTTTGTCTGGT
MV_Leader_as	21-1	CTATCCTTACCCAACTTTGTTTGGT
MV_Leader-s	1-21	ACCAAACAAAGTTGGGTAAGGATAG
M-BstEII-as	4871 -4801	gttgGGTCACCtcggtc
F-BstEII-s	4801 - 4817	gaccgaGGTGACCcaac
MV_H4_as	8458 - 8458	ATCCTTCAATGGTGCCCACTCGGGA
MVH_forward	8385 - 8406	CAGATGACAAGTTGCGAATGGA
Trailer_MV-as	15874 - 15894	ACCAGACAAAGCTGGG AATAGAAAC

Table S2: Primers used to sequence the MV plasmids. Numbers in the nomenclature indicate the nucleotide position according to the MV IC-B sequence (accession no.: AB016162.1).

**Name**	**sequence (5´--->3´)**
T7-Promotor-as	CCCTATAGTGAGTCGTATT
Leader-MV-N-s	ACCAAACAAAGTTGGGTAAGGATAG
MV-N-s/Leader	ACCAAACAAAGTTGGGTAAGG
N-Start-s	ATGGCCACACTTTTGAGG
MV-N-Q-s	TATYGAAGTGCAAGAYCCTGAGG
Roche-MV-N-s	GGGTCTTGCTCGCAAAGG
MV-N-Q-as	CCTTCTTAGCTCYGAATCAGCTG
Roche-MV-N-as	CCTTCTTAGCTCCGAATCAGC
MV-NLC-s	TTAGGGCAAGAGATGGTGAGG
MV-NLC-as	ATCTCTGAAACAAGCCTTGC
P-vor-Start-as	CGGCTCCAGTCGTGGG
P-Start-s	ATGGCAGAAGAGCAGGC
MV-P1	ACTCCAATCCAGAGGCAACAAC
MV-P2	TTCGGGTGTCCACTCCTGTATC
P-Stop-as	CTACTTCATTATTATCTTCATCAG
P-nach-Stop-s	CTACAGCTCAACTTACCTGC
MV-M-SalI-s	ACCCCATGCCAGTCGAC
MV-M-SalI-as	GTCGACTGGCATGGGGT
MV-M-vor-Start-s	TGATTGCCTCCTAAGTTCCACA

MV-M-Start-s	ATGACAGAGATCTACGATTTC
MV-M-Mitte-s	CCTGCCCTTAGGTGTTGGTAG
MV-M-Mitte-as	CCGTTATCCGAAAGACGGGT
MV-M-vor-ende-s	GGTTTTTGCACTTGGTGGGA
MV-M-vor-ende-as	ATCACGTCGTCGTAAATGCG
F-BstEII-s	GACCGAGGTGACCCAAC
M-BstEII-as	GCGGTTGGGTCACCTC
MV-F-vor-Start-s	CACCGGGAATCCCAGAATCA
MV-F-nach-Start-s	CTGCACGAGGGTAGAGATCG
Roche-MV-F-s	ATCAGGCAATTGAGGCAATC
MV-F-Mitte-s	ATTGGCTGTTCAGGGTGTCC
Roche-MV-F-as	GACACCCTGAACAGCCAATATC
MV-F-Mitte-as	TGTCCCTACGTCCAACCTCT
MV-FLA-fwd	GGTTTATCGAGCACTAGCAT
MV-FLA-rev	GACATACCAACTTGTTCTCC
F-PacI-s	CGGTAGTTAATTAAAACTTAGGGTG
F-PacI-as	CACCCTAAGTTTTAATTAACTACCG
MVH-fwd	CAGATGACAAGTTGCGAATGGA
H-SpeI-s	GCATACCCACTAGTGTGAAATAG
H-SpeI 19nt-s	GATGTCACCCAGACATCAG
H-SpeI-as	CTATTTCACACTAGTGGGTATGC
L-Start-s	ATGGACTCGCTATCTGTCAAC
MV-L 9460-s	CTTAGRAGTTAYCCGGCCCA
MV-L 9742-as	AAACCAAAACAGAAAGGGYTCAA
MV-L 10032-s	TGGTTTCTTCCCTGCACTCG
MV-L 10629-s	GGACAAGGCACTTGCTGCTC
MV-L 10821-as	TTGAACTCAGGGTCATGGAGG
MV-L 11053-s	GGGCCAGTCYYAAAAACCYA
MV-L 11374-as	GGGGCAATGAGGRTCACTYA
MV-L 11670-s	TGAYATTGGCCATCACCTCAA
MV-L 11849-as	CRATATTRCTGCATGCTGCCC
L-NheI-s	CTAGACTGGGCTAGCGAC
L-NheI-as	GTCGCTAGCCCAGTCTAG
MV-L 12571-s	GCAGGGATGGTGCTATTGACA
MV-L 12993-as	TCATCATCACCSKAAGCCCA
MV-L 13167-s	CACAATCTCCAAYGACAATCTCTCA
MV-L 13783-as	YGADGACAACAGCTCACCCA
MV-L 14169-s	GGMAGAGGCTAKGYTATCTCCAGC
MV-L 14406-as	TCATCGTGTGGRGGTCTGAA
MV-L 14962-as	TTTGCCMARGAGYAGAGCCA
MV-L 15114-s	WGAYCTCAARGCTAACCGGC
MV-L 15776-as	TCAGRGCRCTGTATCCGACT
L-Stop-as	TTAATCCTTAATCAGAGCGC
MV-Trailer30-s	ATATATTAAAGAAAACTTTGAAAATACGAA

Figure S1: Vector maps of the plasmids

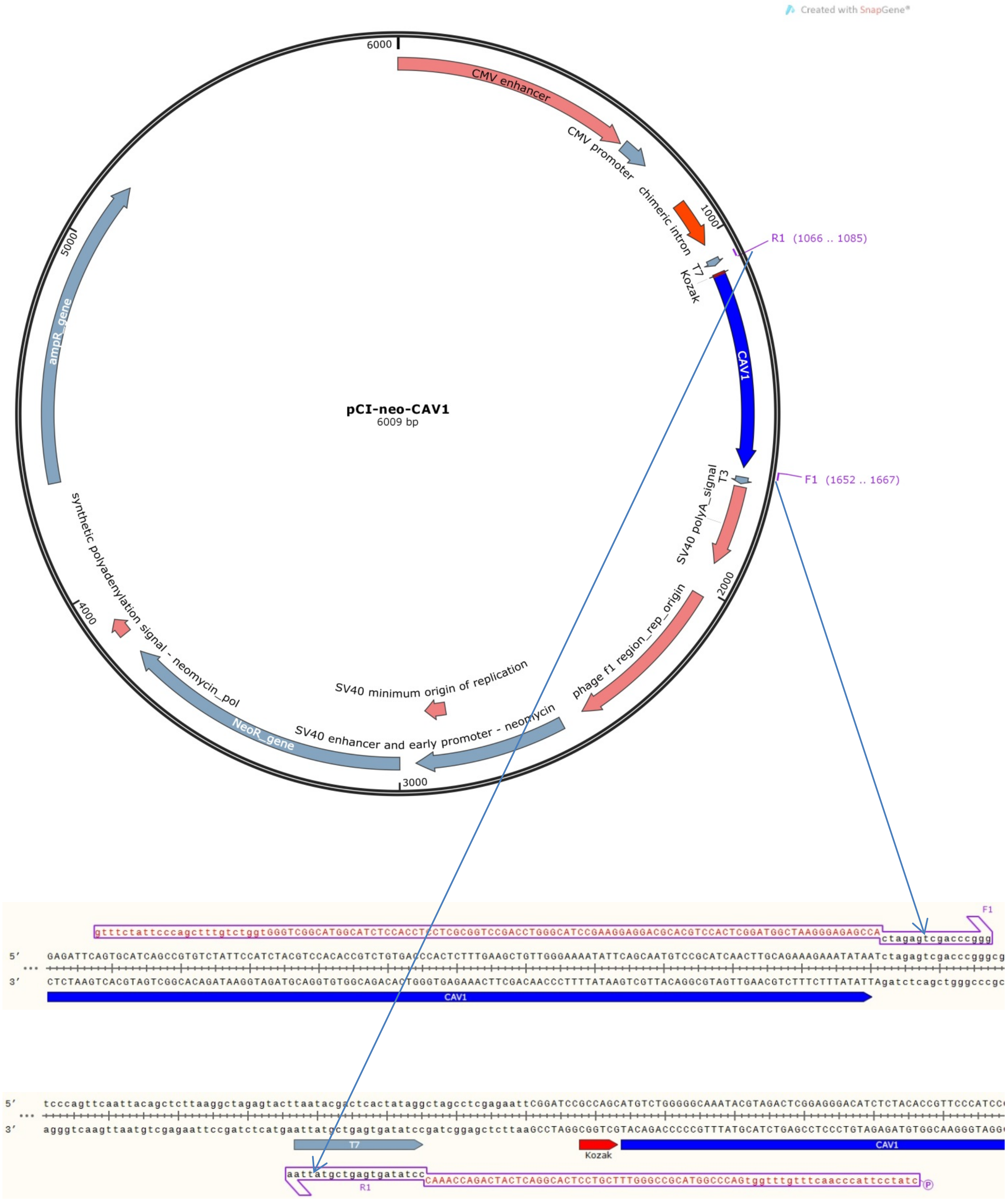

A) **Vector map of the donor plasmid pCi-neo-CAV1 using as a template for the PCR.** Two PCR primers forward primer trailer_HDVrz-pCi-s (F1) and the reverse primer T7_HHrz_leader_as (R1), which contain the MV leader, the trailer, the HHrz and the HDVrz sequences.

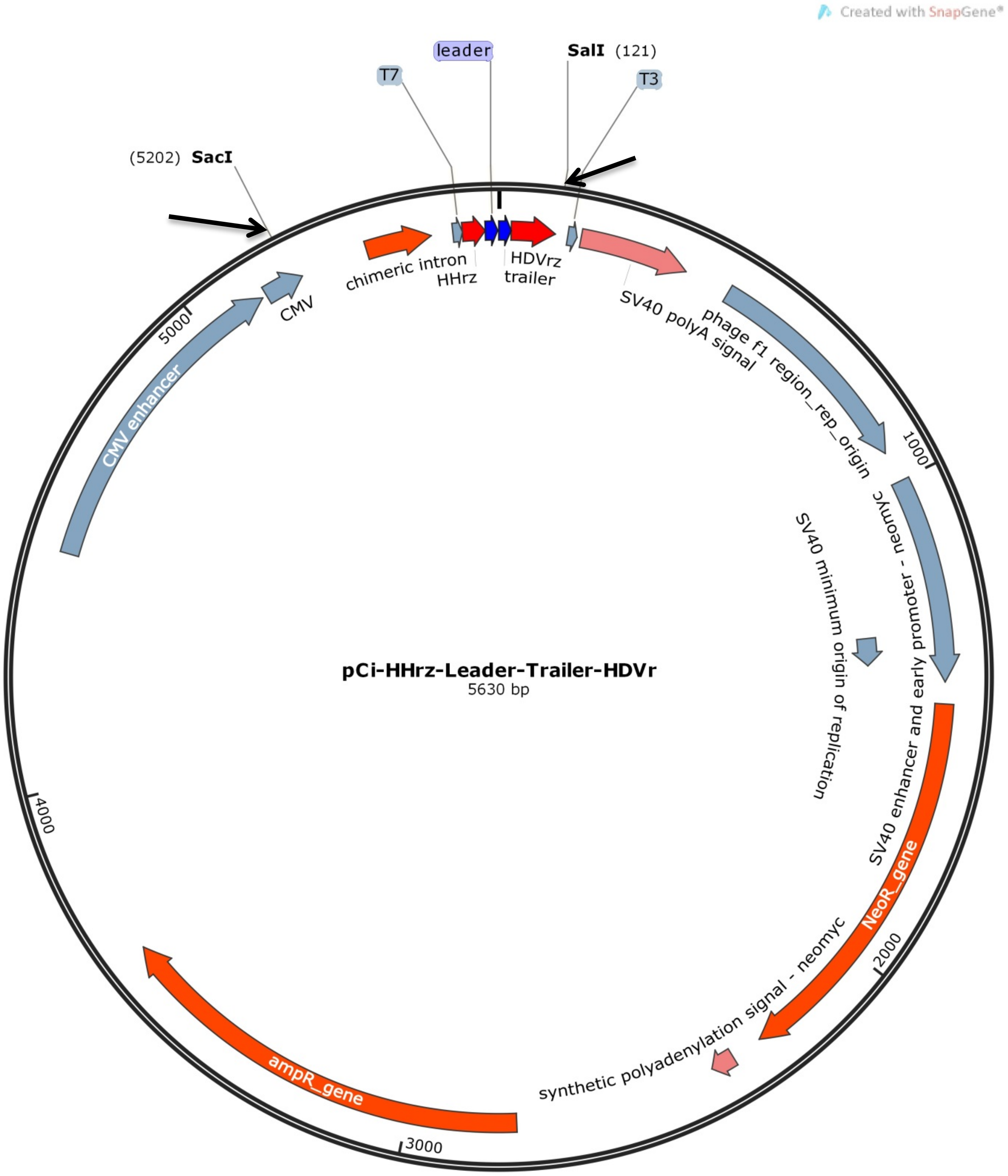

B) **The vector map of the plasmid pCi-HHrz-HDVrz.** The arrows indicate the restriction site for the digestion of the DNA-fragment containing the cassette of the MV sequences.

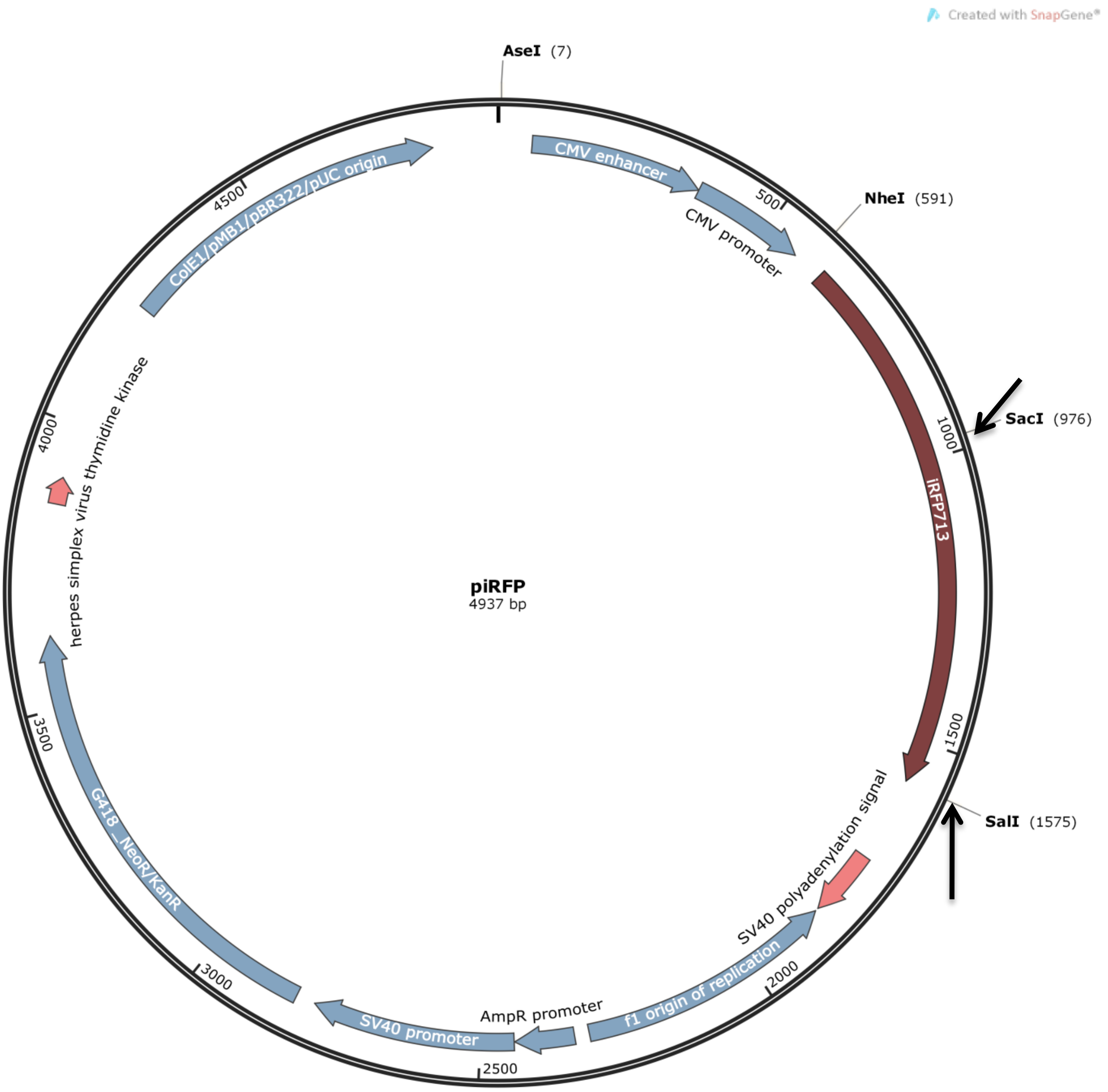

C) **Vector map of the donor plasmid piRFP.** The arrows indicate the restriction sites SacI and SalI for the subcloning into the plasmid pCi-HHrz-HDVrz.

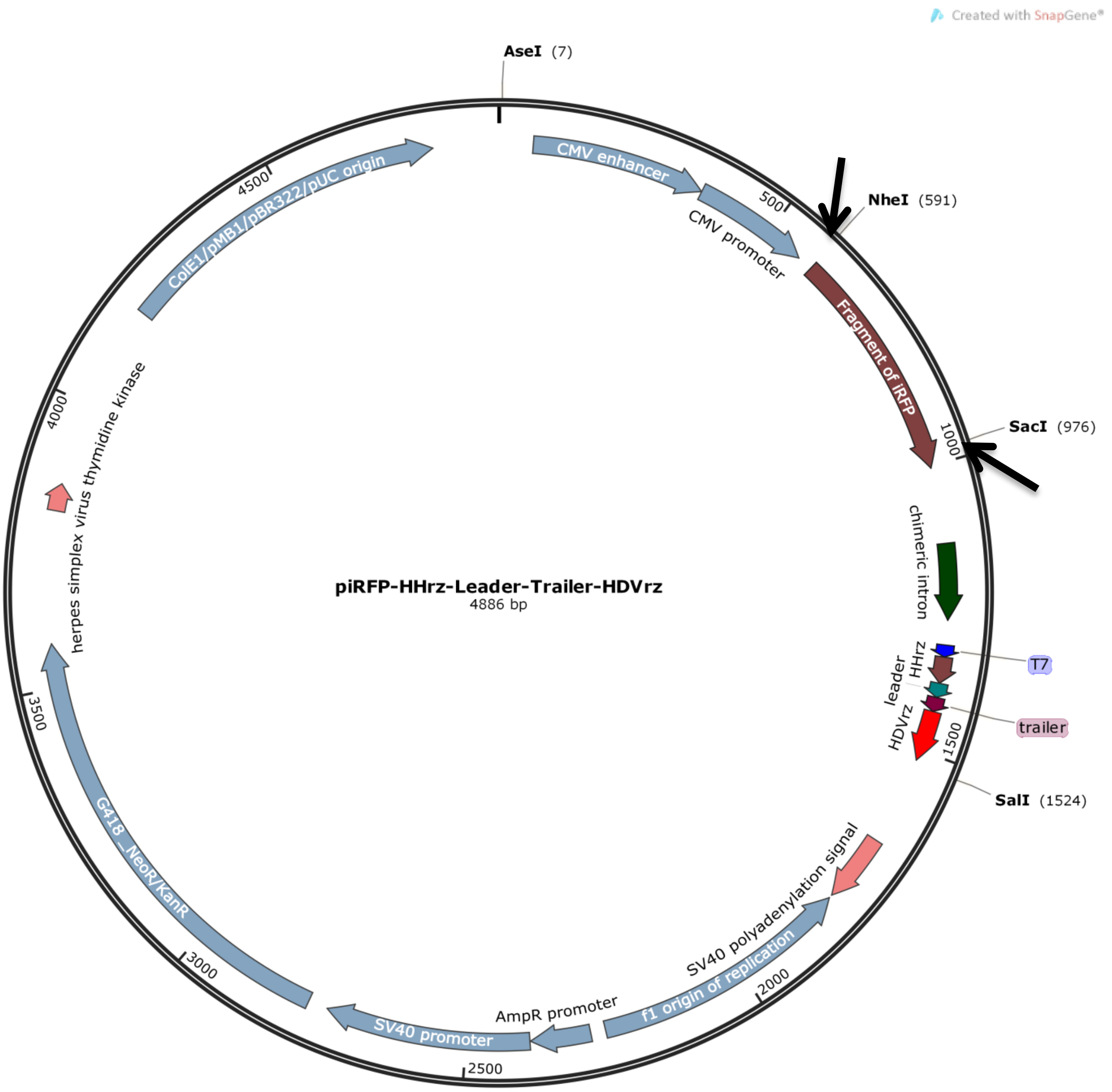

D) **Vector map of the resulting shuttle plasmid piRFP-HHrz-HDVrz.** The arrows indicate the restriction site for the removing the DNA-fragment containing the remaining part of the iRFP gene.

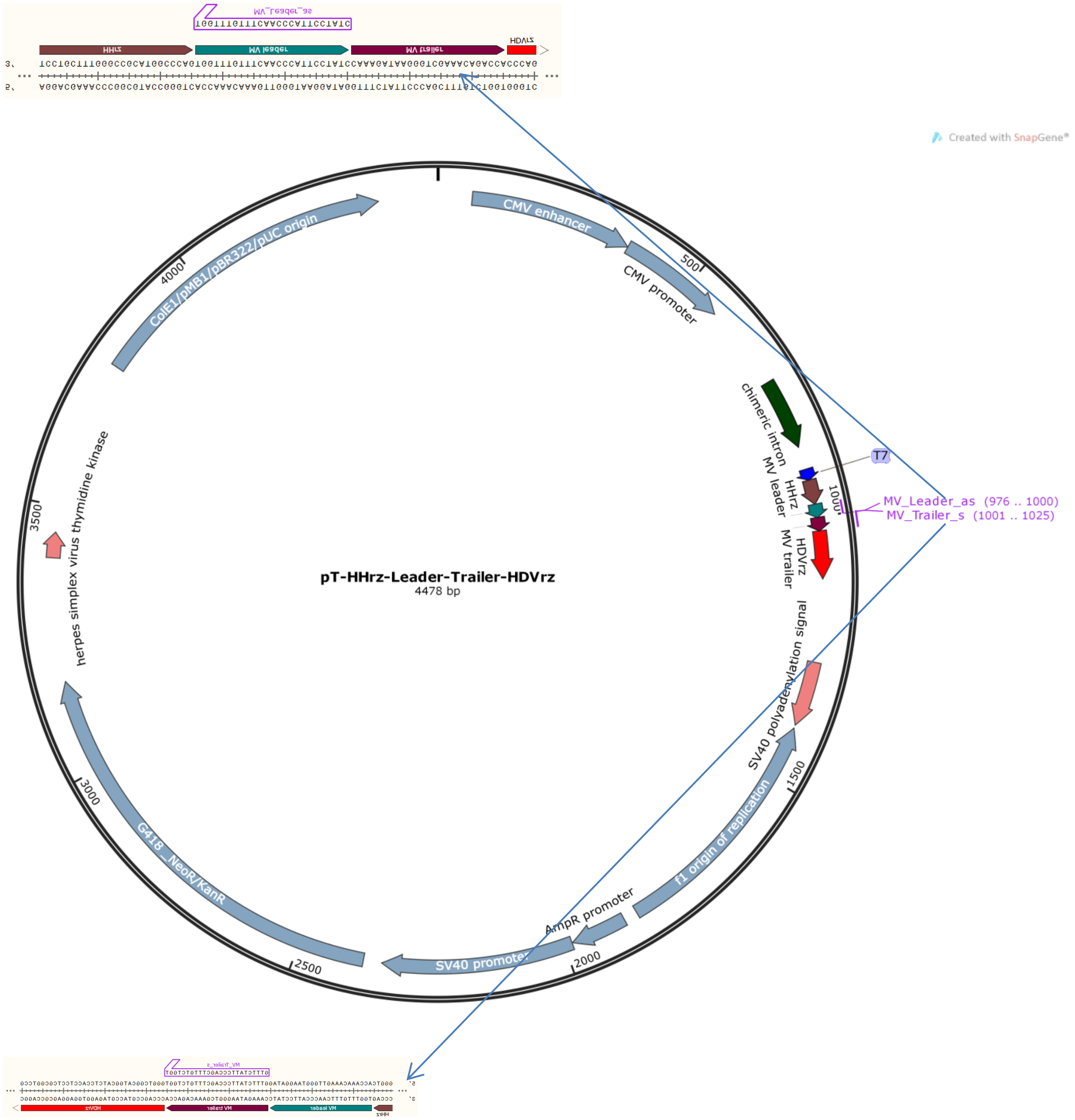

E) **Vector map of the resulting vector backbone plasmid pT-HHrz-HDVrz**

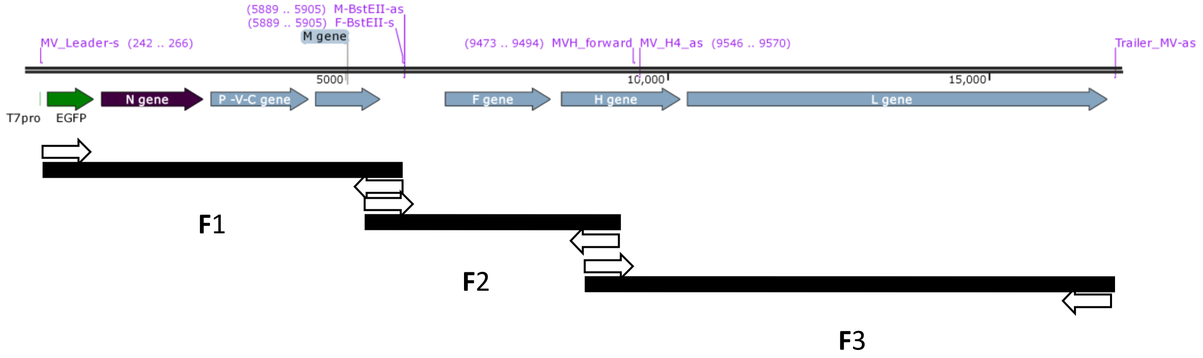

Figure S2: Schematic diagrams of construction of full-length MV cDNA**.** Primer locations and PCRs fragments generated from the MV antigenome template plasmid p(+)MV323-eGFP or p(+)MVAIK-eGFP

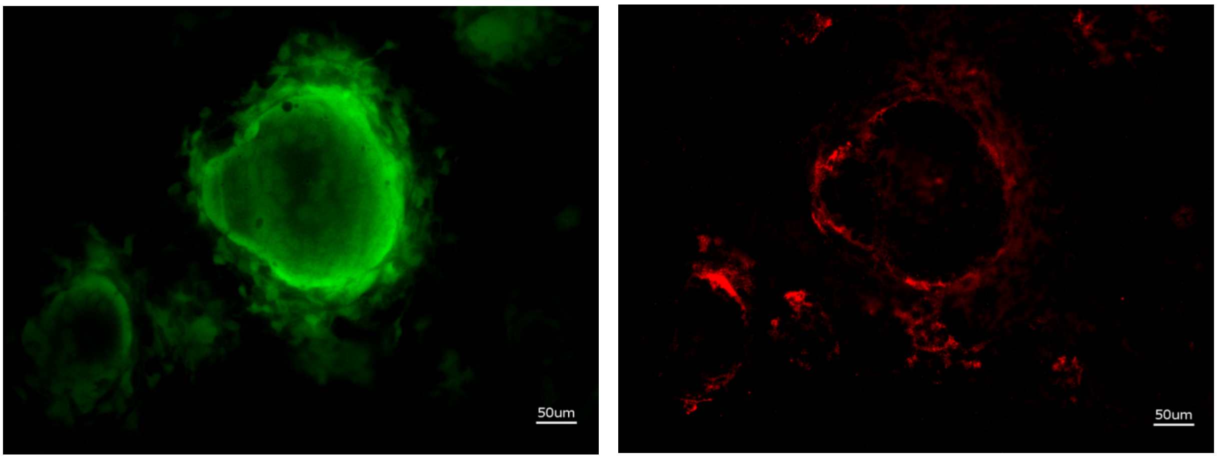

Green fluorescence (eGFP)

Red fluorescence. Cells were immunostained with monoclonal antibogy against MV N protein

Figure S3: Immunohistochemistry for detecting MV N protein. Vero/hSLAM cell monolayers grown on glass coverslips were infected with MV and immunofluorescent staining with primary antibodies mouse anti MV N and secondary antibodies (Alexa fluor 546 donkey anti-mouse).
